# Exposure to War Prior to Conception: Maternal Emotional Distress Forecasts Sex-Specific Child Behavior Problems

**DOI:** 10.3390/ijerph19073802

**Published:** 2022-03-23

**Authors:** Roseriet Beijers, Anat Scher, Hanit Ohana, Ayala Maayan-Metzger, Micah Leshem

**Affiliations:** 1 Behavioural Science Institute, Radboud University, 6525 XZ Nijmegen, The Netherlands; 2 Donders Institute, Radboud University Medical Center, 6525 AJ Nijmegen, The Netherlands; 3 Department of Counseling and Human Development, The University of Haifa, Haifa 3498838, Israel; anats@edu.haifa.ac.il (A.S.); hanit87@gmail.com (H.O.); 4 Neonatal Department, Sheba Medical Center, Ramat Gan 52621, Israel; ayala.maayan@sheba.health.gov.il; 5 School of Psychological Sciences, The University of Haifa, Haifa 3498838, Israel; micahl@psy.haifa.ac.il

**Keywords:** preconception stress, war, behavior problems, executive functioning, child development

## Abstract

Objectives: Exposure to maternal stress during the prenatal period adversely affects child outcomes. Recent investigations have shifted to an even earlier period, the preconception period, to better understand the role of this formative period in human health and disease. We investigated the links between maternal emotional distress following preconception exposure to war, and child outcomes at age 10. Material and Methods: Before becoming pregnant, mothers were exposed to missile bombardment on the north of Israel in the 2006 war. Mothers who conceived within 12 months after the war were recruited and compared to mothers who conceived during the same period but lived in Israel but outside missile range. During the initial assessment, mothers completed a questionnaire on emotional distress. At 10 years of age, mothers and children (*N* = 68) reported on child socio-emotional outcomes. Results: Multiple regression analyses revealed that, in girls, higher maternal emotional distress following preconception war exposure predicted more internalizing and externalizing behavior problems, and more behavior regulation problems. In boys, maternal emotional distress was not significantly related to outcomes. Conclusion: Maternal emotional distress following preconception exposure to war forecasts sex-specific child behavioral problems as reported by the mother and the child. Though the results warrant cautious interpretation because of the relatively small sample size and differential attrition, our findings add to the small but growing body of research on the consequences of maternal stress exposure prior to conception for the next generation.

## 1. Introduction

Exposure to maternal stress during the prenatal period adversely affects child outcomes throughout the lifespan [1,2,3]. Recent investigations have shifted to an even earlier period, the preconception period, to better understand the role of this formative period on human health and disease [4]. For example, studies on Holocaust survivors and their offspring born after the war indicate intergenerational effects of trauma and associated offspring mental health symptoms [5,6]. Maternal exposure to stressful life events prior to conception, such as the death of a close relative, is associated with elevated risk of infant mortality, preterm birth, and low birth weight [7,8,9,10]. Moreover, maternal symptoms of depression and anxiety during the preconception period forecast more infant sleep and maternal-infant bonding problems [11,12], and bereavement in mothers in the six months prior to conception is associated with increased risk for attention-deficit hyperactivity disorder in male offspring [13]. Because few prospective, longitudinal studies exist on maternal exposure to stress during the preconception period, also referred to as pre-gesttional stress, and its impact on child development, the current study investigates long-term consequences of mothers’ emotional distress following preconception war exposure. 

A study by our group investigated the effects of maternal preconception exposure to war in 3-year-old children [14]. Mothers’ exposure to war-related experience was related to elevated levels of maternal emotional distress. In turn, higher levels of maternal emotional distress before pregnancy forecasted higher levels of maternal separation anxiety, and lower levels of maternal emotional availability and child adaptive behavior. As most human studies are retrospective or focus on birth outcomes and early childhood, little is known of whether the possible associations between preconception stress exposure and child functioning are transient, persistent, or may even progress over time. 

This study builds on the previous study by Shachar-Dadon and colleagues [14] that examined the consequences of preconception exposure to war on child outcomes in early development. The Lebanon war erupted unexpectedly as a missile bombardment on the north of Israel and ceased equally abruptly 33 days later, after which near normal life was restored almost immediately [15,16]. Moreover, the type of threat was primarily circumscribed to missile bombardment; there was little fear of enemy invasion and basic services were minimally disrupted. The circumscribed nature and sharp temporal limits of this tragic war afforded a unique opportunity for a quasi-experimental design isolating an epoch of stress. We hypothesized that preconception exposure to war, and associated higher reported maternal emotional distress, were also related to socio-emotional outcomes in childhood. Moreover, as some studies suggest interactions between preconception stress exposure and offspring sex on development [13,17,18,19], we hypothesized sex differences in the association between preconception war exposure and child outcomes. Before becoming pregnant, mothers in this cohort were exposed to the 2006 Lebanon War between the Lebanese Hezbollah and Israel. In order to generate variability in war exposure experiences, mothers were recruited in the northern region of Israel (that sustained bombardment during the war) and were compared to mothers who conceived during the same period but lived outside missile range (central Israel). 

## 2. Materials and Methods

### 2.1. Participants

In order to generate variability in war exposure experiences, mothers were recruited from two locations (T0): The Western Galilee Hospital in the northern region of Israel (an area that sustained bombardment during the war), and Sheba Medical Center in central Israel (beyond missile range). Mothers were included if they conceived between the first month and the twelfth month after the war, and gave birth to healthy, full-term infants with no apparent developmental difficulties. In order to examine preconception stress associations with child outcomes in normally functioning families, mothers were excluded if they experienced clinical levels of posttraumatic stress disorder (PTSD; *N* = 5). For more information on the PTSD screening procedure and details on the participants from our initial assessment, please see the paper of Shachar-Dadon and colleagues [14]. 

When the children were 3 years old (T1), 107 mother-infant dyads participated in a laboratory-based assessment (for more details on the sample characteristics and procedures, see [14]). When the children were 10 years old (T2), 68 of the dyads could be enlisted from the T1 assessment wave. 

Of the 41 families who did not participate at T2, 11 were not interested and 30 could not be included due to incomplete contact details and/or scheduling difficulties. As a result, the proportions of children followed up in the current study were 52% (30/58) of the group of mothers exposed directly to bombardment and 77% (38/49) of the group of mothers not exposed to bombardment. The only significant difference found was that mothers who participated in the T2 data collection had significantly lower T1 maternal separation anxiety (*M* = 94.86, *SD* = 12.68), compared to mothers who did not participate at T2 [(*M* = 100.57, *SD* = 14.29); t(106) = 2.15, *p* = 0.034]. At T2, T1 maternal separation anxiety scores did not differ in mothers exposed to bombardment (*N* = 30, *M* = 97.17, *SD* = 14.06), and mothers not exposed (*N* = 38; *M* = 93.05, *SD* = 11.35; *p* = 0.186). The demographic characteristics of the sample are presented in Table 1. The study was approved by the Institutional Review Board of the University of Haifa (#174/17).

### 2.2. Procedure 

Initial assessment of the mothers during T0 took place after they had given birth, about a year after the war (*M* = 1.1 years; *SD* = 0.4). Mothers completed a questionnaire on demographics (including birth information), emotional distress following preconception war exposure, prewar stressful events, and postwar stressful events. At T2, families were invited by a letter and telephone call to schedule a home visit. During the home visit, an informed consent form was signed by both mother and child. Then mother and child independently completed a set of digital questionnaires. Mothers reported on their children’s internalizing, externalizing, and behavior regulation problems, and their own perceived stress. Children reported on their internalizing and externalizing problems and attachment security. The interviewer sat with the child to help the child understand the instructions. Children received a small gift, and mothers a gift card (~25$).

### 2.3. Measures

#### 2.3.1. Independent Variable (T0)

*Maternal Emotional Distress following Preconception War Exposure* was measured using a 25-item questionnaire designed for the study, assessing the wartime experience, including anxiety/helplessness and physical reactions [14]. On a 5-point Likert scale ranging from 1 (not at all) to 5 (very much), mothers responded to items such as: “To what extent did you experience feelings of anxiety throughout the war?” A total emotional distress variable was created by summing the z-scores of the anxiety/helplessness and physical reactions subscales (α = 0.91). 

#### 2.3.2. Child Outcomes at Age 10 Years (T2)

*Internalizing and Externalizing Behavior* were measured through both child report and mother report using the Strengths and Difficulties Questionnaire (SDQ; [20]). The child/mother responds to items such as “I/My child get(s) very angry and often lose(s) temper.” Items are rated on a 3-point Likert scale (not true to certainly true). The 25 items were grouped into composite scales: Internalizing Problems (mother-report α = 0.71, child-report α = 0.67), and Externalizing Problems (mother-report α = 0.84, child-report α = 0.73). The general rule of thumb is that a Cronbach’s alpha of 0.70 and above is good, but the Cronbach’s alpha of the child-reported Internalizing Problems subscale is questionable. However, as the Cronbach’s alpha is close to 0.70, and previous research has indicated the value of child report above and beyond the maternal report and, especially in case of internalizing problems [21], we continued analyzing all subscales.

*Behavior Regulation Problems* were measured through maternal report with the Behavior Rating Inventory of Executive Function (BRIEF; [22]). The BRIEF is an 86-item questionnaire. Each item loads onto one of eight scales, which form two broader indices: Behavioral Regulation (inhibit, shift, and emotional control; α = 0.85) and Metacognition (initiate, working memory, plan/organize, environmental organization, and monitor; α = 0.88). For the purpose of this paper, the subscale Behavior Regulation was used. Higher scores represent more problems with behavior regulation. 

*Attachment Security* was measured through child report using the Kerns Security Scale (KSS; [23]). The instrument includes a 15-item, self-report questionnaire, designed to assess children’s perception of security of attachment with their mother. The child responded to items such as “some kids worry that their mom may not be there when they need her, but other kids are sure their mom will be there when they need her.” Items were rated from strongly disagree [1] to strongly agree [5], with higher scores indicating a higher level of security (α = 0.72).

### 2.4. Covariates

The covariates included in the present study are similar to the covariates used in our prior study [14], and derive from the T0 or T2 measurement wave: time interval between the end of the war and conception (T0), prewar stressful events (T0), postwar stressful events (T0), child age during the assessment (T2), maternal age (T2), maternal education in years (T2), and maternal perceived stress at child age 10 years (T2). 

Prewar stressful events were measured by asking the mothers during T0 whether they had experienced any of 11 stressful or traumatic events such as exposure to terror, physical or sexual assault, severe accidents or illness or other life threatening events, or familial or economic stress at any time before the Lebanon war. A composite score of previous stressful events was calculated by summing the number of events reported by the mothers [14]. Postwar stressful events were similarly assessed at T0 by asking about the occurrence of any of 11 stressful or traumatic events after the war and during the pregnancy. A composite score of postwar stressful life events was calculated by summing the number of events reported by mothers [14]. Maternal perceived stress [24] at T2 was assessed using the Perceived Stress 10-item self-report measure of stress experienced across the past 30 days on a 5-point scale ranging from 0 (never) to 4 (very often). Higher scores indicate greater maternal perceived stress (α = 0.82). 

### 2.5. Statistical Analyses 

First, the data were checked for outliers. Descriptive statistics and correlations between the study variables and covariates were calculated. Afterwards, independent T-tests were performed to test group differences between the group of mothers who were directly exposed to bombardment (*N* = 30), and mothers not exposed to bombardment (*N* = 38). Lastly, following Shachar-Dadon and colleagues [14], we tested whether maternal emotional distress following preconception war exposure was associated with child outcomes at age 10, and whether any associations were moderated by child gender. For this, standard multiple hierarchical regression analyses were conducted for the six child outcomes (i.e., mother-reported internalizing, externalizing, and behavior regulation problems; child-reported internalizing and externalizing behavior; and attachment security). The covariates, including child gender, were entered in hierarchical step 1, and the main effect of maternal emotional stress and its interaction with gender was entered in hierarchical step 2. To preserve power, the regression analyses only controlled for covariates that were (marginally) significantly correlated to maternal emotional distress. Please note that with an α of 0.05, a β of 0.80, a sample size of 68, and 5 variables, the G*power 3.1 power calculations indicated that small effects can be detected (≥0.093). As this study was not set out to be a longitudinal study from the start, and because of the long time between T0 and T2, we consider the sample size satisfactory. 

## 3. Results

### 3.1. Descriptive Statistics

Table 2 presents the descriptives of the study variables, comparing the two groups. No outliers were identified. Table 3 presents the bivariate correlations between T0 maternal emotional distress and child outcomes at age 10. More maternal emotional stress following preconception war exposure was significantly related to more child internalizing and externalizing behavior problems at age 10. Next, bivariate correlations between maternal emotional distress and the covariates were estimated. More maternal emotional distress was related to fewer maternal years of education (*r* = −0.34, *p* = 0.01), and more reported prewar traumatic experiences (*r* = 0.22, *p* = 0.07). 

### 3.2. Main Analyses 

To first test for group differences, independent T-tests were performed. While mothers directly exposed to the war experienced more emotional stress following preconception war exposure (T0), no significant differences were found between the two groups on child outcomes (see also Table 2).

To secondly test whether maternal emotional distress following preconception war exposure was associated with child outcomes, multiple hierarchical regression analyses were performed and the results are presented in Table 4. As more maternal emotional distress was related to fewer maternal years of education (*r* = −0.34, *p* = 0.01), and more reported prewar traumatic experiences (*r* = 0.22, *p* = 0.07), these two covariates were included in the regression analyses. While no main effects of maternal emotional distress were found, its interaction with gender predicted several child outcomes, as reported by both mother and the child. 

**Figure 1:**
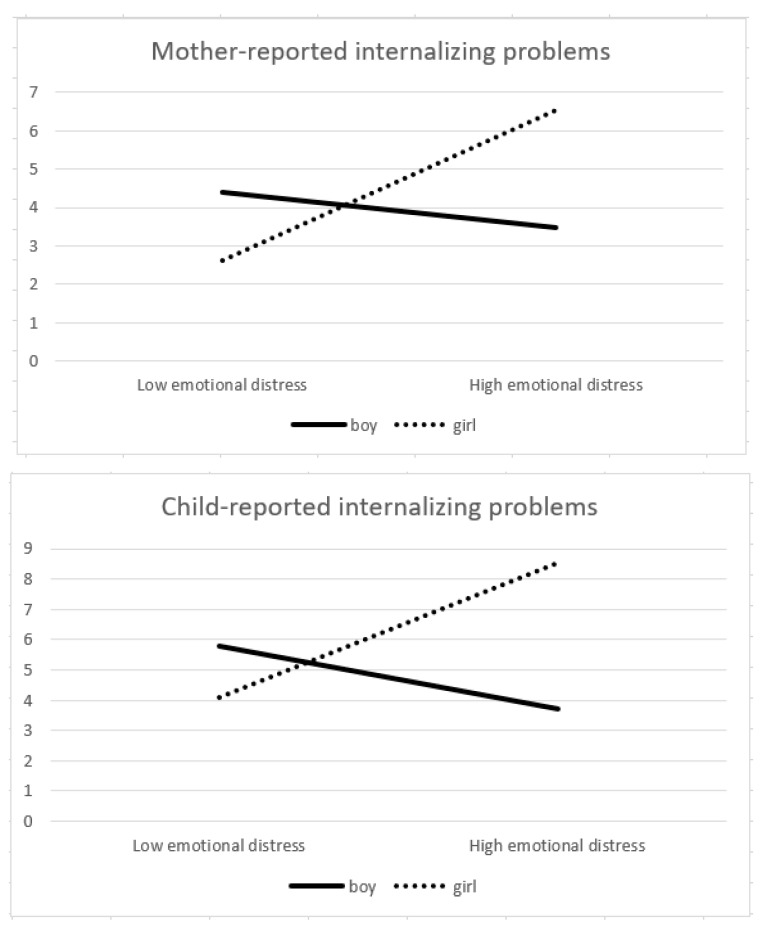

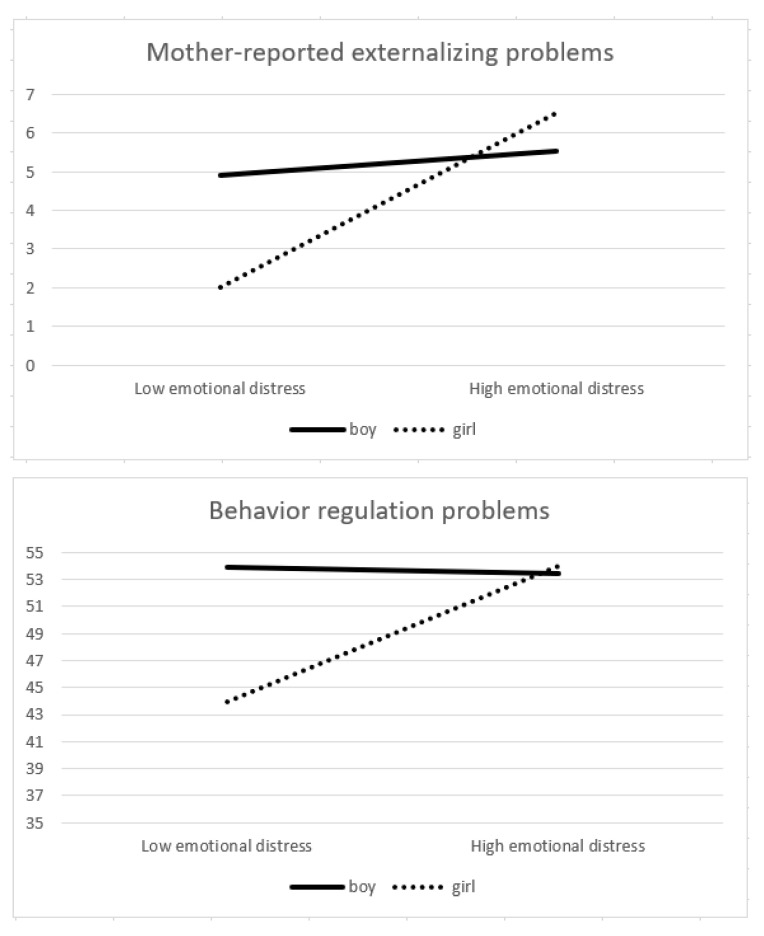
Interaction plots of emotional distress by child gender on child outcomes.

The plots of the (marginally) significant interactions are presented in Figure 1, and slopes were subsequently tested. In girls, maternal emotional distress following preconception war exposure predicted more mother-reported internalizing problems (*t* = 3.12, *p* < 0.01), more externalizing problems (*t* = 3.07, *p* < 0.01), more behavior regulation problems (*t* = 2.71, *p* < 0.01), and more child-reported internalizing problems (*t* = 1.97, *p =* 0.052). Interestingly, in boys, maternal emotional distress did not predict any of the child outcomes. 

## 4. Discussion

The current study investigated whether maternal reported emotional distress following preconception war exposure forecasts poorer socio-emotional outcomes at child age 10. In girls, higher preconception maternal emotional distress predicted more internalizing behavior problems, as reported by both mother and child, and more mother-reported externalizing and behavior regulation problems. Maternal emotional distress was not associated with girls’ reported attachment security. In boys, maternal emotional distress following preconception war exposure was not significantly related to any of the outcomes. Our findings add to the small, but growing, body of research on the implications of maternal stress exposure prior to conception for the next generation and suggest its dependence on child sex. 

Not only animal models support sex-specific effects of preconception stress exposure on offspring neurodevelopment (e.g., [17,18,19,25]), but sex-specific effects of preconception stress exposure are also found in humans. For example, boys (but not girls) born to mothers experiencing the death of a close relative before pregnancy had 47% increased risk of Attention Deficit Hyperactivity Disorder [13]. However, it remains unclear whether male or female offspring are more affected by preconception stress and why. Moreover, in the study of Zaidan, Leshem, and Gaisler-Salomon [26], sex differences in rats were not present at birth but emerged later, and the testing conditions during development moderated the sex-specific links. Thus, the developmental pattern of sex differences might not be static but reflect a dynamic interaction between parental and offspring exposures. 

The question remains what mechanisms underlie the links between maternal emotional distress following preconception war exposure and poorer child socio-emotional outcomes. One possibility is changes in maternal caregiving behavior [5]. In animals, social isolation of female rats immediately after weaning (i.e., a paradigm often used to mimic adversity and elicit stress in rats), decreased the quality of maternal caregiving that these animals later provided to their young (i.e., reduced arched-back nursing, [27]). Within our cohort of children, we found that maternal emotional distress following preconception war exposure was related to higher maternal separation anxiety and lower emotional availability in mother-child interactions at child age 3 [14]. As a large body of work has demonstrated the adverse effects of especially low quality of maternal caregiving behavior on a broad range of child developmental outcomes, including behavior and regulation problems [28,29,30,31], it is possible that maternal separation anxiety and emotional availability mediate the links in girls. As there are animal studies that found maternal preconception stress effects to be already present in the maternal ova as well as at offspring birth [26], and that maternal preconception stress alters offspring prefrontal cortex development without changing maternal care [32], lower quality of maternal caregiving behavior is probably not the (only) mechanism through which preconception stress exposure might affect child outcomes. 

Another mechanism proposed is that exposure to stress prior to conception may spill-over into the gestational period to influence aspects of maternal-placental-fetal biology that are implicated in fetal programming [33]. Research in both animals and humans is providing accumulating evidence that maternal stress during pregnancy can have long-term effects on child development, including socio-emotional outcomes [1,2,3]. If stress continues from the preconception into the prenatal period, exposure to maternal stress during pregnancy may affect the physiology and functioning of the fetus through a network of different pathways and in sex-specific manners [1]. While few studies investigate the contribution of stress exposure during both the preconception and prenatal period, a study found that maternal preconception mental health problems predicted infant emotional reactivity, independently of maternal mental health during the prenatal and postpartum period [34]. Alternative mechanisms likely exist—beyond changes in maternal caregiving behavior and stress spill-over into the prenatal period—to explain the link between preconception stress exposure and offspring outcomes. 

Such alternative mechanisms might implicate that stress exposure prior to conception directly affects the maternal germline with persisting effects on offspring outcomes [6,35,36]. Though studies on the maternal germline are limited, it has, for example, been found that administration of chronic stress to virgin female rats led to 18.5 fold increase in the expression of Corticotropin Releasing Factor type 1 (CRF1) messenger RNA in mature oocytes four days after stress [26]. CRF1 plays a key role in the functioning of the Hypothalamic-Pituitary-Adrenal axis (HPA-axis) and its stress response. While it has been thought for a long time that these epigenetic alterations were completely erased during embryonic development, there is now evidence that some epigenetic marks persist after fertilization [36]. Moreover, as also the fathers in our study were likely affected by the 2006 Lebanon war (some even served in the war), it is also possible that paternal emotional distress following preconception war exposure affected child outcomes through the paternal germline [37,38]. Therefore, we recommend future research to not only investigate maternal, but also paternal, mental health during the preconception period. 

The current study has many strengths, including its reliance on an “experiment by nature design” with uniquely defined stress epoch, longitudinal design, and assessments of behavior problems by two sets of respondents. As maternal emotional distress was associated with both mother-reported and child-reported internalizing problems, the explanation that our results are completely due to a reporter-bias seems less likely. Including two sets of reports is also important as we have already stressed before that one set of reports cannot be presumed to reflect another set of reports (i.e., they are only correlated to some extent, see Table 2), and that particularly in regard to internalizing symptoms—that may not be as readily observed by others compared to externalizing symptoms—it is important to include a child report [21]. However, we have to note that the internal consistency of the child report internalizing symptoms subscale was questionable (Cronbach’s alpha of 0.67). 

There are also additional limitations to note. First, our assessment of T0 maternal emotional distress reported after the war is subject to recall bias. However, when comparing levels of emotional distress between war-exposed mothers and mothers outside missile range, mothers who were directly exposed reported higher levels of emotional distress whereas the reported stressful events before and after the war did not differ. Nevertheless, research should investigate maternal emotional stress during its actual exposure where possible. The differential follow-up rates and the relatively low sample size can also be viewed as limitations. While we were able in the current study to follow up 77% of the children born to mothers not exposed to bombardment, the proportion of children born to mothers directly exposed to bombardment was smaller (i.e., 52%). As the attrition analyses found that mothers who did participate at T2, compared to the mothers who did not participate, had significantly lower T1 maternal separation anxiety, and that we previously showed that more emotional distress following preconception war exposure was related to higher levels of maternal separation anxiety [14], it is possible that the differential attrition rates obscured potential larger or other effects. Furthermore, the relatively low sample size precluded the possibility of investigating whether maternal outcomes at child age 3 mediate the link between maternal emotional distress and the poorer outcomes found in girls at age 10. Moreover, the low sample size precluded the possibility to investigate if the time interval between the end of the war and conception moderated the links found. However, and importantly, finding effects in a small sample after a decade of normality, coupled with the fact that all mothers lived in country with war and that mothers who participated in the current data collection had significantly lower maternal separation anxiety levels at child age 3 than non-participating mothers, might suggest that the effects we found are conservative. 

## 5. Conclusions

The findings indicate that maternal distress following preconception war exposure predicts sex-specific behavioral issues a decade later. As maternal emotional distress following preconception war was not only associated with maternal report of child socio-emotional functioning, but also with child report, our findings seem robust and likely not due to a reporter-bias. Though a natural experiment is constrained by its circumstance, the unique characteristics of this study captured a one-time-only combination of circumstances suggesting that maternal preconception stress should be further investigated as a determinant of child development. In addition, as animal studies indicate that intervention may mitigate effects of preconception stress whether provided between stress and conception or postnatally [18], future research should consider interventions that may mitigate its effects.

## Figures and Tables

**Table 1:** Descriptives of the demographics.

	Mothers Directly Exposed to Bombardment, *N* = 30	Mothers not Exposed to Bombardment, *N* = 38
	*M* (*sd*)	*M* (*sd*)
Girls	63%	42%
Child age in years (T2)	10.02 (0.31)	10.09 (0.35)
Maternal age in years (T2)	41.96 (3.46)	43.93 (4.79)
Maternal education in years (T2)	14.70 (1.44)	15.84 (1.88) **
Number of children (T2)	2.90 (0.55)	2.95 (1.22)
Marital status (T2)	96.7%	86.8%
Gestational age (T0)	39.1 weeks (10.8 days)	39.2 weeks (9.9 days)
Infant birth weight (T0)	3214.83 g (422.47)	3339.74 g (569.96)
Maternal stress (T2)	25.37 (6.43)	23.16 (5.79)
Prewar stressful events (T1)	0.80 (0.41)	0.74 (0.45)
Postwar stressful events (T1)	0.43 (0.50)	0.29 (0.46)

** *p* = 0.01. Independent T-tests indicated that mothers directly exposed to the war had significantly less years of education at T2, and still lived in the same regions.

**Table 2:** Descriptives of the study variables.

	Mothers Directly Exposed to Bombardment, *N* = 30	Mothers not Exposed to Bombardment, *N* = 38
	*M* (*sd*)	*M* (*sd*)
*Predictor (T0)*		
Maternal wartime emotional distress	0.70 (1.81)	−0.55 (1.60) **
*Child Outcomes (T2)*		
Reported by the mother:		
Internalizing problems (SDQ)	4.40 (2.94)	4.08 (3.76)
Externalizing problems (SDQ)	4.77 (4.23)	4.42 (3.87)
Behavioral regulation (BRIEF)	49.87 (9.76)	51.92 (10.63)
Reported by the child:		
Internalizing problems (SDQ)	6.07 (3.30)	4.95 (3.11)
Externalizing problems (SDQ)	6.80 (3.68)	6.24 (3.55)
Attachment security (Kerns)	3.21 (0.41)	3.27 (0.34)

** *p* = 0.01. Independent T-tests indicated that mothers experienced more emotional stress following preconception war exposure (T0). No significant differences were found between the two groups on child outcomes.

**Table 3:** Correlations between the study variables.

	1	2	3	4	5	6	7	8	9	10	11	12	13	14	15
1. Maternal emotional distress (T0)															
Mother-reported child outcomes (T2)															
2. Internalizing problems	0.24 *														
3. Externalizing problems	0.32 **	0.46 **													
4. Behavior regulation problems	0.22	0.65 **	0.68 **												
Child-reported child outcomes (T2)															
5. Internalizing problems	0.11	0.42 **	0.23	0.30 *											
6. Externalizing problems	0.17	0.30 *	0.53 **	0.44 **	0.51 **										
7. Attachment insecurity	0.03	−0.27 *	−0.31 *	−0.28 *	−0.42 **	−0.52 **									
Covariates															
8. Child gender (1 = boy, 2 = girl)	0.09	0.05	−0.13	−0.27 *	0.09	−0.09	−0.00								
9. Time interval war and conception	0.01	−0.13	−0.09	0.01	0.08	−0.07	−0.06	−0.05							
10. Prewar stressful events (T0)	0.22 ^†^	0.23 ^†^	0.21 ^†^	0.22 ^†^	0.25 *	0.22 ^†^	−0.20 ^†^	0.02	−0.01						
11. Postwar stressful events (T0)	0.13	0.12	−0.10	−0.00	0.13	−0.05	−0.01	0.04	−0.18	−0.17					
12. Child age at assessment (T2)	−0.01	0.01	−0.02	−0.09	−0.06	−0.01	0.14	0.17	−0.37 **	0.29 *	0.09				
13. Maternal age (T2)	−0.09	−0.11	−0.17	−0.19	−0.13	−0.12	0.07	0.05	0.01	0.05	−0.23 ^†^	0.08			
14. Maternal education in years (T2)	−0.34 **	−0.08	−0.06	0.04	−0.02	−0.10	0.08	−0.30 *	0.07	−0.03	0.10	0.18	−0.14		
15. Maternal perceived stress (T2)	0.17	−0.00	0.10	0.16	0.18	0.07	−0.03	0.06	−0.09	0.11	0.02	−0.16	−0.28 *	−0.29 *	

^†^ = *p* < 0.10, * *p* = 0.05, ** *p* = 0.01.

**Table 4:** Results of the multiple hierarchical regression analyses predicting child outcomes as reported by the mother.

		B	β	*p*	R^2^ Model	F_change_	R^2^_change_
**Mother-reported child outcomes**							
**Internalizing problems**							
Step 1	prewar stressful events	1.805	0.227	0.059	0.059		
	maternal education in years	0.047	0.025	0.846			
	child gender ^1^	0.213	0.032	0.792			
Step 2	maternal emotional distress	−0.232	−0.122	0.472	0.197	5.319	0.138
	emotional distress X gender	1.236	0.457	**0.006**			
**Externalizing problems**							
Step 1	prewar stressful events	1.644	0.175	0.142	0.074		
	maternal education in years	0.013	0.006	0.963			
	child gender	−1.288	−0.162	0.179			
Step 2	maternal emotional distress	0.162	0.073	0.668	0.196	4.722	0.122
	emotional distress X gender	1.001	0.314	**0.056**			
**Behavior regulation problems**							
Step 1	prewar stressful events	4.947	0.207	0.080	0.123		
	maternal education in years	0.251	0.044	0.727			
	child gender	−5.648	−0.278	**0.021**			
Step 2	maternal emotional distress	−0.126	−0.022	0.895	0.218	3.733	0.094
	emotional distress X gender	2.718	0.334	**0.040**			
**Child-reported child outcomes**							
**Internalizing problems**							
Step 1	prewar stressful events	2.01	0.267	0.033			
	maternal education in years	0.086	0.048	0.718			
	child gender ^1^	0.592	0.093	0.457	0.070		
Step 2	maternal emotional distress	−0.333	−0.186	0.293			
	emotional distress X gender	0.891	0.348	**0.042**	0.133	2.265	0.063
**Externalizing problems**							
Step 1	prewar stressful events	1.739	0.207	0.097			
	maternal education in years	0.330	0.164	0.219			
	child gender	−0.449	−0.063	0.614	0.065		
Step 2	maternal emotional distress	0.026	0.013	0.941			
	emotional distress X gender	0.717	0.251	0.141	0.127	2.199	0.062
**Attachment security**							
Step 1	prewar stressful events	−0.212	−0.244	0.057	0.046		
	maternal education in years	0.022	0.105	0.443			
	child gender	0.015	0.021	0.872			
Step 2	maternal emotional distress	0.057	0.276	0.132	0.082	1.233	0.036
	emotional distress X gender	−0.067	−0.225	0.196			

^1^: child gender: 1 = boy, 2 = girl.

## Data Availability

Raw data were generated at Haifa University. Derived data supporting the findings of this study are available from the corresponding author [R.B.] on request.
